# Clinical, Conventional CT and Radiomic Feature-Based Machine Learning Models for Predicting ALK Rearrangement Status in Lung Adenocarcinoma Patients

**DOI:** 10.3389/fonc.2020.00369

**Published:** 2020-03-20

**Authors:** Lan Song, Zhenchen Zhu, Li Mao, Xiuli Li, Wei Han, Huayang Du, Huanwen Wu, Wei Song, Zhengyu Jin

**Affiliations:** ^1^Department of Radiology, Peking Union Medical College Hospital, Chinese Academy of Medical Sciences and Peking Union Medical College, Beijing, China; ^2^4+4 MD Program, Chinese Academy of Medical Sciences and Peking Union Medical College, Beijing, China; ^3^Deepwise AI Lab, Deepwise Inc., Beijing, China; ^4^Department of Epidemiology and Biostatistics, Institute of Basic Medicine Sciences, Chinese Academy of Medical Sciences and School of Basic Medicine, Peking Union Medical College, Beijing, China; ^5^Department of Pathology, Peking Union Medical College Hospital, Chinese Academy of Medical Sciences and Peking Union Medical College, Beijing, China

**Keywords:** lung neoplasms, radiomics, tomography, X-ray computed, anaplastic lymphoma kinase, gene mutation

## Abstract

**Objectives:** To predict the anaplastic lymphoma kinase (ALK) mutations in lung adenocarcinoma patients non-invasively with machine learning models that combine clinical, conventional CT and radiomic features.

**Methods:** This retrospective study included 335 lung adenocarcinoma patients who were randomly divided into a primary cohort (268 patients; 90 ALK-rearranged; and 178 ALK wild-type) and a test cohort (67 patients; 22 ALK-rearranged; and 45 ALK wild-type). One thousand two hundred and eighteen quantitative radiomic features were extracted from the semi-automatically delineated volume of interest (VOI) of the entire tumor using both the original and the pre-processed non-enhanced CT images. Twelve conventional CT features and seven clinical features were also collected. Normalized features were selected using a sequential of the F-test-based method, the density-based spatial clustering of applications with noise (DBSCAN) method, and the recursive feature elimination (RFE) method. Selected features were then used to build three predictive models (radiomic, radiological, and integrated models) for the ALK-rearranged phenotype by a soft voting classifier. Models were evaluated in the test cohort using the area under the receiver operating characteristic curve (AUC), accuracy, sensitivity, and specificity, and the performances of three models were compared using the DeLong test.

**Results:** Our results showed that the addition of clinical information and conventional CT features significantly enhanced the validation performance of the radiomic model in the primary cohort (AUC = 0.83–0.88, *P* = 0.01), but not in the test cohort (AUC = 0.80–0.88, *P* = 0.29). The majority of radiomic features associated with ALK mutations reflected information around and within the high-intensity voxels of lesions. The presence of the cavity and left lower lobe location were new imaging phenotypic patterns in association with ALK-rearranged tumors. Current smoking was strongly correlated with non-ALK-mutated lung adenocarcinoma.

**Conclusions:** Our study demonstrates that radiomics-derived machine learning models can potentially serve as a non-invasive tool to identify ALK mutation of lung adenocarcinoma.

## Introduction

Non-small cell lung cancer (NSCLC), especially lung adenocarcinoma, is the leading cause of cancer-related deaths worldwide (1, 2). The occurrence of fused anaplastic lymphoma kinase (ALK) gene in NSCLC patients is ~5% in western countries, but ALK mutations have become the second most significant molecular mutations in the regimen of NSCLC treatment following epidermal growth factor receptor (EGFR) mutations (2–6). The positivity rate of ALK is similar in the Asian population with NSCLC (4.9%) and is higher in those with lung adenocarcinomas (6.03%) (7). The accurately screening of ALK mutation patients has thus become a pivotal step in treating NSCLC.

Traditional molecular tests for detecting ALK rearrangements including fluorescence *in situ* hybridization (FISH) and immunohistochemistry (IHC) are limited in the detection of genetic mutations and monitoring of therapeutic effects. Firstly, the required biopsies or surgical resection may not be attainable for vulnerable and advanced cancer patients. In addition, recent studies have reported a 30–87.5% intra-tumoural genetic heterogeneity rate for ALK fusions in NSCLCs, which challenges the accuracy of traditional ALK fusion tests based on tissues from a routine biopsy procedure (8–10). Moreover, given the low occurrence of ALK mutations among NSCLCs, the purchasing of the devices and antibodies required for such molecular tests were cost-inefficient for both hospitals and patients. Therefore, a non-invasive, convenient, and more reliable procedure for detecting ALK mutations is necessary.

Computed tomography (CT) is widely used to diagnose lung cancer. Recent studies have identified some CT imaging features that are associated with ALK gene rearrangements, including central tumor location, lobulated margin, solidity, pleural effusion, and distant metastasis (11–14). However, the evaluation of these conventional CT features depends heavily on the radiologist's experience and is time-consuming. Radiomics is a computer-based approach that has been widely applied in the diagnosis of lung neoplasm as well as the prediction of survival and gene mutations in lung cancer (15–18). It could help radiologists to identify additional information about tumor phenotype that is distinct from conventional findings of CT images (15, 16, 19–21). So far, the efficacy of radiomics in predicting the ALK gene in lung adenocarcinoma is still unknown. Therefore, the aim of our study is to (1) investigate the role of radiomic features in the prediction of ALK rearrangement status in lung adenocarcinomas, and (2) examine whether or not the addition of conventional CT characteristics and clinical data can improve the performance of the predictive model.

## Materials and Methods

### Patient Population

**Figure 1:**
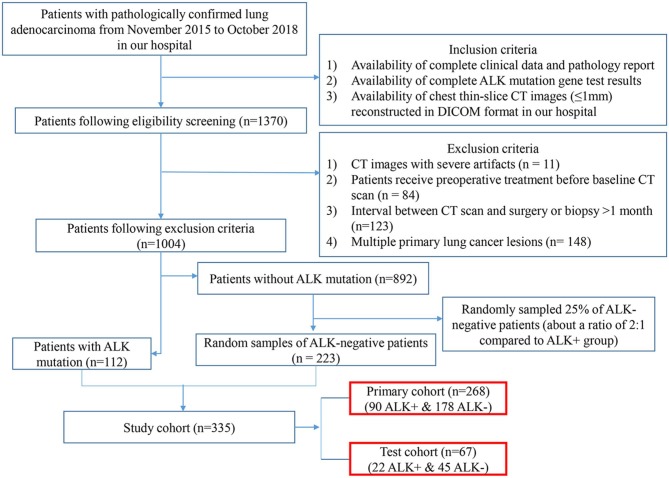
Eligibility and exclusion criteria of the study. The flowchart depicts the process of patient enrolment, including eligibility, and exclusion criteria of the study. The numbers in parentheses are the numbers of patients. ALK, anaplastic lymphoma kinase; DICOM, Digital Imaging and Communications in Medicine.

This retrospective study reviewed a total of 1,370 consecutive patients with pathologically confirmed lung adenocarcinoma by surgery or biopsy at our hospital from November 2015 to October 2018. The inclusion criteria were as follows: (1) availability of complete clinical data; (2) complete ALK mutation gene test results; (3) availability of complete thin-slice chest CT images (≤ 1 mm) reconstructed in Digital Imaging and Communications in Medicine (DICOM) format. The exclusion criteria were as follows: (1) CT images with severe artifacts; (2) patients receiving treatment before CT examinations; (3) interval between CT examination and surgery or biopsy >1 month; (4) multiple primary lung cancers. According to these criteria, 1,004 patients (112 ALK-positive and 892 ALK-negative) were eligible for the investigation. We randomly sampled 25% of the ALK-negative patients for enrolment in our study. Finally, 335 patients (112 ALK+ patients and 223 ALK– patients) were enrolled in this study. Twenty percent of the cases were randomly selected from the ALK+ and ALK– patients, respectively, to build an independent test cohort (67 cases, 22 ALK+ and 45 ALK–; median age, 57 years; range, 34–78 years) while the remaining being the primary cohort (268 cases, 90 ALK+ and 178 ALK–; median age, 58 years; range, 26–83 years). The flowchart of the eligibility and exclusion criteria is shown in Figure 1. The tumor lesions were all solitary. This retrospective study was approved by our institutional review board, and the need for informed patient consent was waived.

In regards to molecular profiles, the Ventana ALK (D5F3) CDx assay (the antibody clone D5F3 with OptiView amplification and OptiView detection, Ventana Medical Systems Inc.) coupled to a BenchMark XT automated staining instrument (Roche/Ventana Medical Systems Inc.) was used to test ALK fusion genes on the formalin-fixed paraffin-embedded tissues. Tissues were from either biopsy or surgical procedures. Specimens were scored binarily as positive if strong granular cytoplasmic brown staining was present in tumor cells. The international consensus guideline has now regarded the Ventana IHC method as an alternative to the conventional FISH test (22). For the IHC score for ALK that was near the borderline, FISH tests were conducted to make the final decision.

### Image Acquisition and Lesion Segmentation

Non-enhanced chest CT scans of 335 patients were carried out from the lung apex to the lung base using multi-detector CT (MDCT) scanners from Siemens (Somatom Definition Flash or Somatom Force; Forchheim, Germany), General Electric (Discovery CT750 HD; Milwaukee, WI), Philips (IQon CT; The Netherlands) or Toshiba (Aquilion 64; Tokyo, Japan) at the end of inspiration. Breath-hold training was carried out before each examination. The following scanning parameters were used: slice thickness/slice increment 1 mm (Somatom Definition Flash, Somatom Force and IQon CT) or 0.625 mm (Discovery CT750 HD) or 0.5 mm (Aquilion 64); rotation time 0.5 s (Somatom Definition Flash, Somatom Force, Aquilion 64, IQon CT) or 0.6 s (Discovery CT750 HD); pitch 0.984 (Aquilion 64, Discovery CT750 HD) or 1.2 (Somatom Definition Flash, Somatom Force, IQon CT); matrix 512 × 512; high and standard resolution algorithms; tube voltage 120 kVp, tube current adjusted automatically.

**Figure 2:**
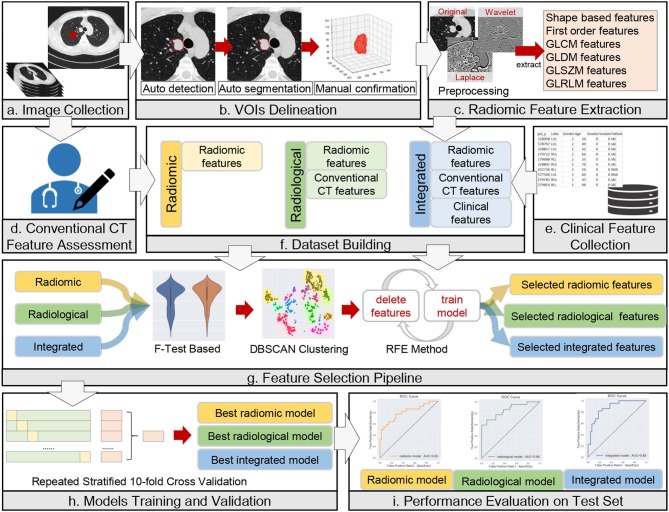
Workflow of data analysis. The workflow illustrates the radiomic, radiological, and integrated modeling and analysis workflow with one example of a CT image and tumor segmentation. **(a)** A male lung adenocarcinoma patient, 44 years old. **(b)** Auto-detection, segmentation, and manual confirmation of the targeted lesion. The red square in the first image mimics the detection process. The initial regions of interest (ROIs) are generated in this step. **(c–e)** Description of the process of collection of radiomic, conventional CT and clinical features. **(f–i)** Illustrations of dataset building, feature selection, model training and validation, and model evaluation, respectively.

The anonymized thin-slice DICOM format non-enhanced CT images were imported into the Dr. Wise research platform, on which the lesions were automatically delineated with automatic pulmonary nodule detection and segmentation algorithms (23). The detection model was a two-stage network that integrated both image and feature pyramids for nodule detection. The segmentation model was built based on the recurrent convolutional neural networks, and the attention map was used to improve model performance. Both the detection model and segmentation model were trained on a combination of public and in-house datasets (details in Supplementary Information 1.1). The results were confirmed and modified on axial images slice by slice with lung window settings (width, 1,200 HU, level, −500 HU) by two thoracic radiologists with 3 and 14 years of diagnostic imaging experience, without knowledge of pathological report information or other information. The volume of interest (VOI) was drawn according to the tumor-lung interface, excluding vascular, bronchus, atelectasis, and other adjacent normal tissues as much as possible. The whole process of the data analysis workflow is depicted in Figure 2.

### Collection of Clinical Data and Evaluation of Conventional CT Features

Clinical data were collected through electronic medical records, including the following seven characteristics: age, sex, smoking history, smoking index, clinical stage, distal metastasis, and pathological invasiveness of the tumor. The clinical stage was determined according to the eighth edition of the American Cancer Society guidelines for NSCLC staging (24). The pathological subtypes of adenocarcinoma *in situ* (AIS), minimally invasive adenocarcinoma (MIA) and invasive adenocarcinoma (IAC) were assessed according to the latest International Multidisciplinary Classification of Lung Adenocarcinoma guidelines (25).

All thin-slice CT images were evaluated by 2 radiologists (with 14 and 3 years of chest CT interpretation experiences) who were blinded to each subject's clinical data. Decisions on CT findings were reached by consensus. Twelve CT morphological features were assessed, including maximum diameter, mean CT attenuation, lesion location, involved lobe, density, margin, cavity, calcification, pleural retraction sign, pleural effusion, pericardial effusion, and local lymphadenopathy. The definitions and scoring rules of the clinical features and conventional CT features are described in Supplementary Table 1.

### Radiomic Feature Extraction

**Figure 3:**
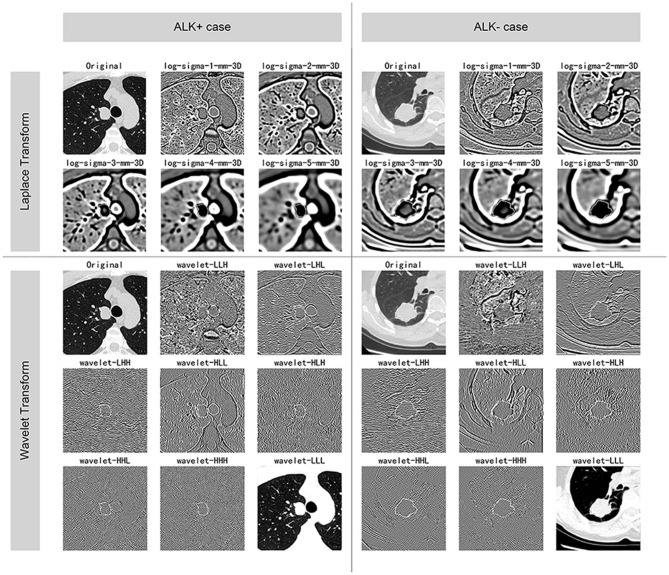
Illustration of the pre-processing methods. The figure displays the VOIs of selected ALK+ and ALK– invasive adenocarcinoma cases after each procedure of the image pre-processing methods. The ALK-positive case was a 44-years-old male patient, and the ALK-negative case was a 60-years-old female patient. Both of the lesions were solid and light lobulated.

The images were resampled to a pixel spacing of 1.0 mm in three anatomical directions to offset the interference caused by the inconsistent spatial resolution. Then high-pass and low-pass wavelet filters or Laplacian of Gaussian (LoG) filters with different σ parameters were employed to pre-process the original image. The results of the pre-processed images from one ALK+ case and one ALK– case after each pre-processing technique are illustrated in Figure 3. A total of 1,218 radiomic features were extracted from the segmented three-dimensional VOIs of the tumor on non-enhanced CT images and the pre-processed images. The features quantified the phenotypic characteristics of the tumors and were divided into three groups: first-order features, shape features, and texture features. The texture features included gray level co-occurrence matrix (GLCM), gray level size zone matrix (GLSZM), gray level run length matrix (GLRLM), and gray level dependence matrix (GLDM) features. All steps above were performed using the PyRadiomics tool (version 2.1.0). The demonstration of filtering and the detailed explanations of all radiomic features can be found in the Supplementary Informations 1.2, 1.3.

### Feature Selection and Development of Predictive Models

We grouped the features into three sets—the radiomic set (radiomic features), the radiological set (radiomic features + conventional CT features), and the integrated set (radiomic features + conventional CT features + clinical features). Each of the three sets was selected and then used to develop the radiomic model, radiological model and the integrated model in the primary cohort individually. To maximize the generalization ability of our model and to reduce the bias of the performance evaluation, the entire feature selection and model training procedure was fed into a repetitive (10 runs) 10-fold cross-validation using the primary cohort. The discriminative score for each patient was obtained from averaging the final predictive probabilities of the classifiers. The area under the curve (AUC) was calculated from the assembled probability. The optimized hyper-parameters of the feature selection and model training procedure were obtained by a grid search that maximized the AUC of the repetitive 10-fold cross-validation. After the hyper-parameters were determined, the model was re-trained using the entire primary dataset and the performance on the test cohort was viewed as the estimation of the true performance of our model. The above procedures were performed by the Scikit-learn software package (Version: 0.20.3) on the Dr. Wise research platform.

Before the feature selection procedure, the features were pre-processed to fit the machine-learning algorithm, including Min–Max scaling for all numerical features and one-hot encoding for categorical features. We used a three-step sequential procedure that was consisted of the F-test-based method, the density-based spatial clustering of applications with noise (DBSCAN) method (26), and the recursive feature elimination (RFE) method (27). The F-test-based method examined the difference of means of each feature between the ALK-rearranged group and the wild-type group, and features with smaller *P*-values were retained. In the unsupervised DBSCAN method, the paired features with high Pearson correlation coefficients were clustered. The border of the cluster was defined by the radius of the cluster (eps) and the minimum number of points within the cluster (min sample size). Within each cluster, only the feature with the smallest *P*-value in the previous method was remained at this step. Besides, non-clustered features were also retained. The logistic regression (LR) based RFE method was used as the last selection process, in which we set the regularization intensity to 0.5 and penalty as L1. For each iteration, two features with the least coefficients were pruned until the desired number of features to select was eventually reached.

A soft voting classifier was used to build the predictive model. In this classifier, the average of the predicted probabilities of being ALK+ trained with the LR model and that trained with the decision tree (DT) model was used as the final predictive probability of the predictive model.

### Statistical Analysis

The differences in all variables between ALK-positive group and ALK-negative group were assessed using Mann-Whitney *U*-test or independent samples *t*-test for continuous variables, and chi-square test or Fisher's exact test for categorical variables as appropriate. This step was performed with SPSS Statistics 20.0 (IBM Corporation, NY, USA). The predictive models were analyzed using the receiver operating characteristics (ROC) curve. The AUC, 95% confidence interval (CI) for AUC, accuracy, sensitivity, and specificity were calculated. The cut-off discriminative score to differentiate ALK-mutated patients and ALK wild-type patients was determined by maximizing the Youden index in the training process. The above analyses were performed by the Scikit-learn software package (Version: 0.20.3) and the Matplotlib package (Version 3.1.0) on the Dr. Wise research platform. Lastly, the DeLong test was used for pairwise comparisons among the three models using MedCalc software (Version 19.0.2). A two-sided *P* < 0.05 was considered statistically significant throughout the study.

## Results

### Clinical and Conventional CT Features

Among the entire cohort, 269 (80.3%) patients underwent surgical procedures and 66 (19.7%) underwent diagnostic biopsies. The results of clinical features in the primary and the test cohort are listed in Table 1. The rates for the number of ALK-mutated patients vs. ALK-negative patients in the primary and the test cohort were both close to 1:2. All clinical characteristics but the smoking history (*P* = 0.028) for patients in the primary and the test cohort showed no statistical difference.

**Table 1:** Clinical characteristics of ALK– and ALK+ lung adenocarcinoma patients in the primary and test cohort.

**Characteristics**	**Primary cohort**				**Independent test cohort**			
	**Total/%**	**ALK–/%**	**ALK+/%**	***P*-value^**b**^**	**Total/%**	**ALK–/%**	**ALK+/%**	***P*-value^**b**^**
Age (years^a^)	57 ± 10 (26–83)	59 ± 10 (28–83)	54 ± 10 (26–73)	<0.001^*^	57 ± 11 (34–78)	59 ± 10 (40–78)	54 ± 10 (34–76)	0.116
Sex								
Male	113**/**42.2	76**/**42.7	37**/**41.1	0.804	26**/**38.8	19**/**42.2	7**/**31.8	0.412
Female	155**/**57.8	102**/**57.3	53**/**58.9		41**/**61.2	26**/**57.8	15**/**68.2	
Smoking history								
Never	182**/**67.9	111**/**62.4	71**/**78.9	<0.001^*^	50**/**74.6	33**/**73.3	17**/**77.3	0.578
Current	74**/**27.6	65**/**36.5	9**/**10.0		10**/**14.9	8**/**17.8	2**/**9.1	
Former	12**/**4.5	2**/**1.1	10**/**11.1		7**/**10.4	4**/**8.9	3**/**13.6	
SI (pack-years)								
SI ≤ 10	208**/**77.6	127**/**71.3	81**/**90.0	0.002^*^	55**/**82.1	36**/**80	19**/**86.4	0.581
10 <SI < 20	9**/**3.4	8**/**4.5	1**/**1.1		2**/**3.0	2**/**4.4	0**/**0	
SI ≥ 20	51**/**19.0	43**/**24.2	8**/**8.9		10**/**14.9	7**/**15.6	3**/**13.6	
Pathology								
AIS	12**/**4.5	10**/**5.6	2**/**2.2	0.109	1/1.5	1/2.2	0**/**0	0.410
MIA	22**/**8.2	18**/**10.1	4**/**4.4		7/10.4	6/13.3	1**/**4.5	
IAC	234**/**87.3	150**/**84.3	84**/**93.3		59/88.1	38/84.4	21**/**95.5	
DM (−)	244**/**91.0	174**/**97.8	70**/**77.8	<0.001^*^	58/86.6	43/95.6	15**/**68.2	0.004^*^
DM (+)	24**/**9.0	4**/**2.2	20**/**22.2	(Fisher)	9/13.4	2/4.4	7**/**31.8	(Fisher)
Clinical stage								
I	176**/**65.7	141**/**79.2	35**/**38.9	<0.001^*^	46**/**68.7	36**/**80	10**/**45.5	0.002^*^
II	32**/**11.9	16**/**9.0	16**/**17.8		4**/**6.0	3**/**6.7	1**/**4.5	
III	15**/**5.6	7**/**3.9	8**/**8.9		6**/**9.0	4**/**8.9	2**/**9.1	
IV	45**/**16.8	14**/**7.9	31**/**34.4		11**/**16.4	2**/**4.4	9**/**40.9	

The data are displayed as n/%, except where otherwise noted. No significant difference exists between the primary and test cohort for all demographic characteristics (P > 0.05) but the smoking history (P = 0.028).

a Mean ± standard deviation (range).

b ALK– group vs. ALK+ group.

* P < 0.05.

*ALK, anaplastic lymphoma kinase; AIS, adenocarcinoma in situ; MIA, minimally invasive adenocarcinoma; IAC, invasive adenocarcinoma; SI, smoking index; DM, distant metastasis; Fisher, Fisher's exact test*.

In the primary cohort, the patients in the ALK-positive group were significantly younger than those in the ALK-negative group (*P* < 0.001). In addition, more patients in the ALK mutation group had advanced lung cancers (stages III and IV), distant metastases and no smoking history than those in the ALK wild-type group. In terms of conventional CT features (see Table 2), ALK mutated lesions were found to have larger size and hyper-attenuation, and tended to be solid, lobulated, with more prevalence of pleural effusion, pericardial effusion, and local lymphadenopathy (*P* < 0.01). There was a higher percentage of central tumors in the ALK+ group than in the ALK– group (*P* = 0.008), although the peripheral lesions were more common within each group. Cavities were slightly more frequent in lesions with ALK mutations (*P* = 0.039).

**Table 2:** Conventional CT features of ALK– and ALK+ lung adenocarcinoma patients in the primary and test cohort.

**Features**	**Primary cohort**				**Independent test cohort**			
	**Total/%**	**ALK–/%**	**ALK+/%**	***P*-value^**b**^**	**Total/%**	**ALK–/%**	**ALK+/%**	***P*-value^**b**^**
mDia. (mm)^a^	19 ± 16	18 ± 15	21 ± 23	0.007^*^	22 ± 19	18 ± 15	26 ± 22	0.089
CT attenuation (HU)^a^	−214 ± 476	−397 ± 455	−7 ± 197	<0.001^*^	5 ± 289	−35 ± 409	26 ± 38	0.001^*^
Location								
Central	43**/**16.0	21**/**11.8	22**/**24.4	0.008^*^	12**/**17.9	4/8.9	8/36.4	0.014^*^
Peripheral	225**/**84.0	157**/**88.2	68**/**75.6		55**/**82.1	41/91.1	14**/**63.6	(Fisher)
Lobe								
RUL	78**/**29.1	57**/**32.0	21**/**23.3	0.280	16**/**23.9	12/26.7	4**/**18.2	0.274
RML	14**/**5.2	10**/**5.6	4**/**4.4		0**/**0	0**/**0	0**/**0	
RLL	58**/**21.6	40**/**22.5	18**/**20.0		17**/**25.4	11**/**24.4	6**/**27.3	
LUL	65**/**24.3	43**/**24.2	22**/**24.4		19**/**28.4	14**/**31.1	5**/**22.7	
LLL	51**/**19.0	27**/**15.2	24**/**26.7		13**/**19.4	8**/**17.8	5**/**22.7	
Mixed	2**/**0.7	1**/**0.6	1**/**1.1		2**/**3.0	0**/**0	2**/**9.1	
Density								
pGGO	83**/**31.0	74**/**41.6	9**/**10.0	<0.001^*^	10**/**14.9	10**/**22.2	0**/**0	<0.001^*^
pSolid	69**/**25.7	51**/**28.7	18**/**20.0		25**/**37.3	22**/**48.9	3**/**13.6	
Solid	116**/**43.3	53**/**29.8	63**/**70.0		32**/**47.8	13**/**28.9	19**/**86.4	
Margin								
Spiculated	115**/**42.9	86**/**48.3	29**/**32.2	0.004^*^	35**/**52.2	29**/**64.4	6**/**27.3	0.009^*^
Lobulated	120**/**44.8	67/37.6	53**/**58.9		28**/**41.8	13**/**28.9	15**/**68.2	
Smooth	33**/**12.3	25**/**14.0	8**/**8.9		4**/**6.0	3**/**6.7	1**/**4.5	
Cavity (–)	244**/**91.0	167**/**93.8	77**/**85.6	0.039^*^	63**/**94.0	42**/**93.3	21**/**95.5	1.000
Cavity (+)	24**/**9.0	11**/**6.2	13**/**14.4		4**/**6.0	3**/**6.7	1**/**4.5	(Fisher)
Calcification (–)	256**/**95.5	170**/**95.5	86**/**95.6	1.000	60**/**89.6	41/91.1	19**/**86.4	0.675
Calcification (+)	12**/**4.5	8**/**4.5	4**/**4.4	(Fisher)	7**/**10.4	4/8.9	3**/**13.6	(Fisher)
Plu. retraction (–)	133**/**49.6	85**/**47.8	48**/**53.3	0.388	26**/**38.8	18**/**40.0	8**/**36.4	0.774
Plu. retraction (+)	135**/**50.4	93**/**52.2	42**/**46.7		41**/**61.2	27**/**60.0	14**/**63.6	
Plu. effusion (–)	237**/**88.4	168**/**94.4	69**/**76.7	<0.001^*^	57**/**85.1	44/97.8	13**/**59.1	<0.001^*^
Plu. effusion (+)	31**/**11.6	10**/**5.6	21**/**23.4		10**/**14.9	1/2.2	9**/**40.9	(Fisher)
Per. effusion (–)	258**/**96.3	178**/**100	80**/**88.9	<0.001^*^	58**/**86.6	43**/**95.6	15**/**68.2	0.004^*^
Per. effusion (+)	10**/**3.7	0**/**0	10**/**11.1	(Fisher)	9**/**13.4	2**/**4.4	7**/**31.8	(Fisher)
Lymph. (–)	205**/**76.5	158**/**88.8	47**/**52.2	<0.001^*^	48**/**71.6	38**/**84.4	10**/**45.5	0.001^*^
Lymph. (+)	63**/**23.5	20**/**11.2	43**/**47.8		19**/**28.4	7**/**15.6	12**/**54.5	

The data are displayed as n**/**%, except where otherwise noted.

a Median ± interquartile interval.

b ALK– group vs. ALK+ group.

* P < 0.05.

*ALK, anaplastic lymphoma kinase; mDia., maximum diameter; RUL, right upper lobe; RML, right middle lobe; RLL, right lower lobe; LUL, left upper lobe; LLL, left lower lobe; pGGO, pure ground-glass opacity; pSolid, partial solid; Plu., pleural; Per., pericardial; Lymph., lymphadenopathy; Fisher, Fisher's exact test*.

### Features Selection and Model Construction

**Figure 4:**
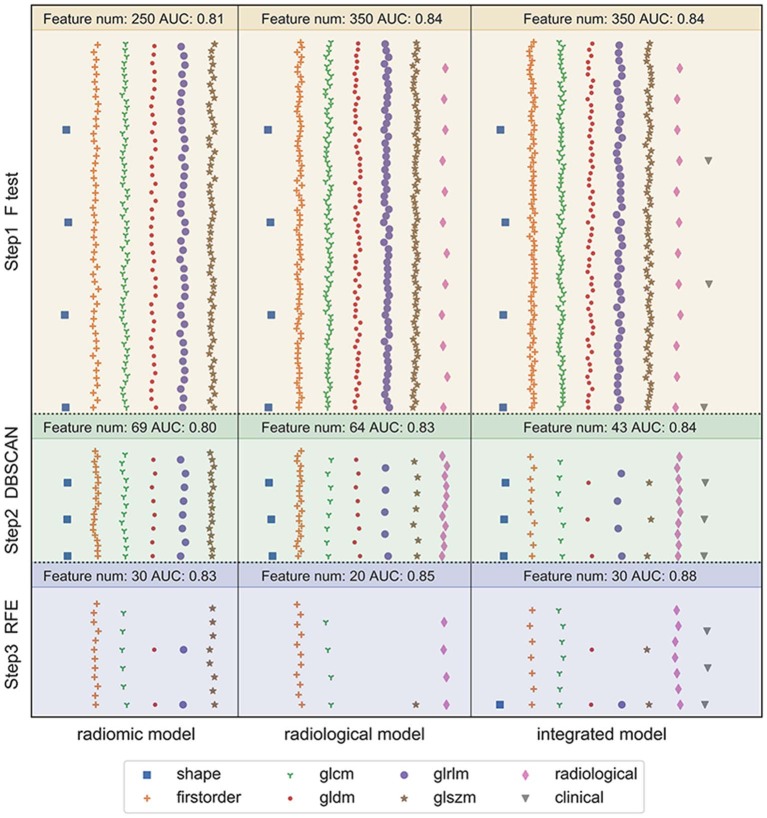
Illustration of the feature selection procedure in the three models. Each vertical panel exhibits the selection process for each of the three predictive models. Each symbol indicates a different type of feature. The number of selected features along with the optimal AUC obtained at each selection step was shown at the top of each sub-panel. In the radiomic model, 1,218 extracted radiomic features were used to begin the selection. In the radiological model, the initial features included 12 conventional CT features and 1,218 radiomic features. In the integrated model, seven clinical characteristics were added in addition to the 12 conventional CT features and 1,218 radiomic features. The features were selected to maximize the AUC of the predictive model at the final step.

**Figure 5:**
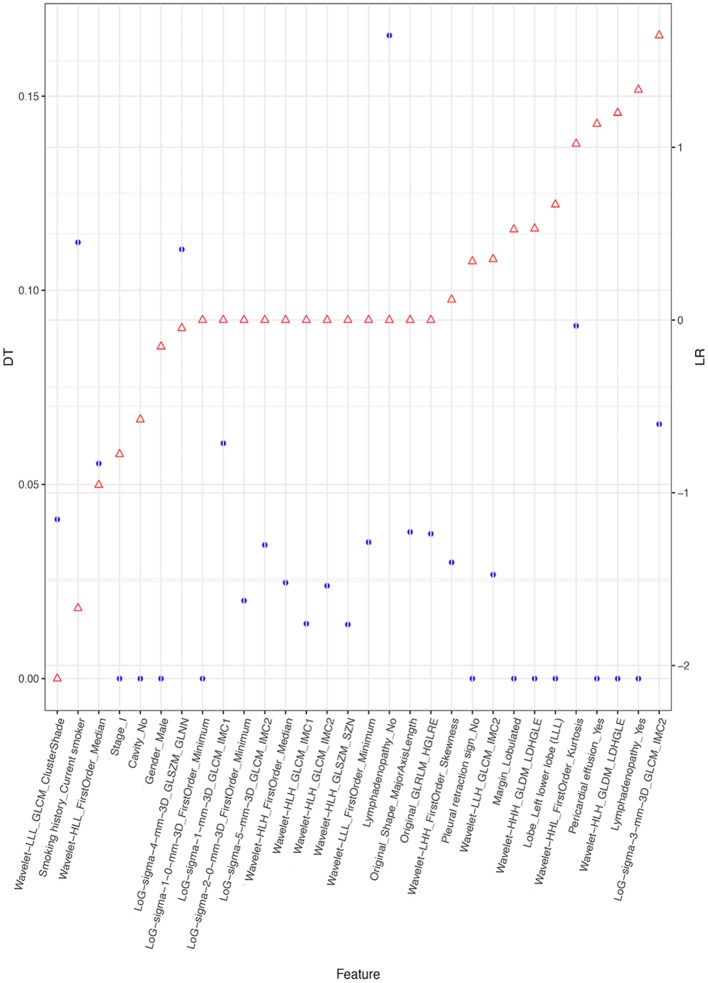
Selected features and their coefficients in the integrated model. The blue dots indicate the coefficients in the DT model. They denote the decrease of the Gini index when such a feature is used in the DT model. A higher DT value suggests a more significant influence. The red dots represent the beta coefficient in the LR model. Since all features were rescaled before the selection procedure, these coefficients are equivalent to the normalized LR coefficients. A higher positive LR coefficient (right side of the figure) suggests a stronger relationship between the feature and ALK mutation, and a higher negative LR coefficient (left side of the figure) suggests a stronger relationship between the feature and ALK-negative status.

Figure 4 depicts the procedure of feature selection sequences. The final models contained 30, 20, and 30 features in the radiomic, radiological, and integrated models, respectively. The hyper-parameters associated with each selection method in each predictive model are displayed in Supplementary Table 2. The majority of selected radiomic features throughout the three prediction models were first-order features and texture features. The only shape-based feature (Original_Shape_MajorAxisLength) was used in the integrated model. In the radiomic model, features that had positive non-zeros coefficients in both DT and LR model were Original_Firstorder_90Percentile, Original_Firstorder_Maximum, and Wavelet-LHH_GLDM_LDHGLE. For conventional CT features, pericardial effusion, local lymphadenopathy, lobulated margin, and the absence of pleural retraction sign were selected in both the radiological and integrated model as being correlated with ALK-rearranged status. The integrated model also adopted no cavity and left lower lobe lesions, as shown in Figure 5. The favorable clinical features for ALK-negative status (negative LR coefficients) were current smoking, early clinical stage (stage I) and male sex. The list of the selected features and their associated coefficients in DT and LR model are illustrated in Supplementary Tables 3–5.

### Evaluation of Models and Comparison of Predictive Model Performance

**Figure 6:**
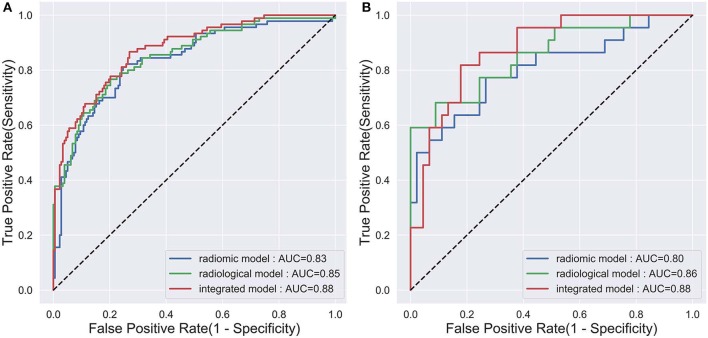
The ROC curves of the three prediction models that indicate ALK mutation status. **(A)** The validation set in the primary cohort; **(B)** the test cohort.

**Figure 7:**
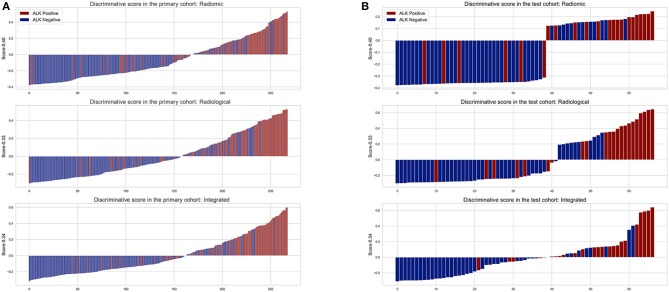
The discriminative scores of the three predictive models in the primary **(A)** and test cohort **(B)**. The discriminative score for each patient is the average of the final predictive probabilities in the LR and DT classifier. The columns above the horizontal axis represent tumors that were predicted to be ALK+, while the columns below the horizontal axis represent the opposite. The color indicates the golden truth of each tumor.

The diagnostic performance of each model is shown in Table 3 and the results of ROC curve analysis are shown in Figure 6. The optimal thresholds that maximized the Youden index for the radiomic model, radiological model, and integrated model were 0.40, 0.33, and 0.34, respectively. The prediction results of each model when validating the cross-validation cohort and in the test cohort are shown in Figure 7. We predicted the lesion as ALK-positive if the discriminative score for that lesion was higher than the threshold in each model, and as ALK-negative if otherwise.

**Table 3:** Diagnostic performance of each model in the primary cohort and test cohort.

**Model name**	**Primary cohort**					**Independent test cohort**			
		**AUC (95% CI)**	**ACC**	**SEN**	**SPE**	**AUC (95% CI)**	**ACC**	**SEN**	**SPE**
Radiomic	Train	1.00 (0.99–1.00)	1.00	1.00	0.99	0.80 (0.69–0.89)	0.73	0.73	0.73
	Validation	0.83 (0.79–0.88)	0.76	0.70	0.80				
Radiological	Train	1.00 (0.99–1.00)	1.00	1.00	1.00	0.86 (0.75–0.93)	0.75	0.68	0.78
	Validation	0.85 (0.80–0.89)	0.78	0.78	0.78				
Integrated	Train	1.00 (0.99–1.00)	1.00	1.00	0.99	0.88 (0.77–0.94)	0.79	0.82	0.78
	Validation	0.88 (0.83–0.91)	0.79	0.78	0.80				

*In the primary cohort, the performance index of each model in the training and the validation set were displayed separately. The radiomic model contained the selected radiomic features only. The radiological model contained the selected conventional CT features in addition to the radiomic features. The integrated model contained the selected radiomic features, conventional CT features and clinical characteristics. AUC, area under the receiver operating characteristic curve; ACC, accuracy; SEN, sensitivity; SPE, specificity*.

In the primary cohort, the performances of the three predictive models in the training set were close to perfect. In the validation set, the integrated model achieved the best performance (AUC = 0.88). A statistically significant difference in AUC was found between the integrated model and the radiomic model with the DeLong test (*P* = 0.01), but not between the integrated model and the radiological model (*P* = 0.1) or the radiological model and radiomic model (*P* = 0.25). In the test cohort, although the integrated model also showed the highest AUC (0.88) among the three predictive models, no statistical difference was found between any of the two models using DeLong test (*P* = 0.35 for radiomic vs. radiological; *P* = 0.29 for radiomic vs. integrated; *P* = 0.66 for radiological vs. integrated).

## Discussion

In this study, we developed an integrated model that combined radiomic features, clinical data and conventional CT features (AUC = 0.88, accuracy = 0.79, sensitivity = 0.82, and specificity = 0.78 in the independent test cohort) for differentiating ALK mutations in lung adenocarcinoma patients. During this process, we identified that Original_Firstorder_90Percentile, Original_Firstorder_Maximum, and Wavelet-LHH_GLDM_LDHGLE were significant and robust radiomic features associated with ALK mutation. These features reflect abstract information from the distribution of pixel intensity and the texture morphology that cannot be detected with the naked eyes. We also found that the addition of conventional CT features to the radiomic model did not increase the model's efficacy, yet the clinical data, in combination with conventional CT features were able to significantly enhance the performance of the prediction model in the cross-validation set. Among the clinical features, smoking history was the most powerful factor to differentiate ALK mutated lung adenocarcinomas from the non-ALK mutated ones. Moreover, our study optimized the performance of models by using the automatic lesion segmentation techniques, involving features from filtered images, and adopting a soft voting classifier.

The model with radiomic features alone in our study reached an AUC of 0.83, which is not inferior to other previously established clinical models that were based on conventional CT features (also named as morphological or semantic CT features) and patients' clinical information (11, 28, 29). This suggests the strong efficacy of radiomics as tools to identify ALK-mutated tumours' phenotypic patterns on CT scans in lung adenocarcinoma patients. The construction of the radiomic model was purely based on features within the first-order and texture categories, which suggests that the intensity distribution of tumors was a strong predictive factor for ALK genetic mutation. This is consistent with findings in other radiomic studies (15, 16, 20). Among the selected radiomic features, Original_Firstorder_90Percentile, Original_Firstorder_Maximum, and Wavelet-LHH_GLDM_LDHGLE were the most significant and robust features associated with ALK mutations, which reflect tumour's intensity and textural features surrounding and within the high-intensity CT voxels. This finding could be related to the revelation that ALK+ lung tumors were more likely to be solid mass (13, 28, 30, 31).

In our study, conventional CT evaluations contained tumour's surrounding information that was typically not represented by radiomic features of tumor itself. In our radiological model, three out of the four selected conventional CT features reflected the relationship between tumor and its surrounding tissue. They were pericardial effusion, local lymphadenopathy, and no pleural retraction sign. These features and their correlations with ALK mutations have been identified in previous literature (14, 28, 30). These pathological changes around the ALK-mutated tumor may result from the infiltration of tumor cells, suggesting the more invasiveness nature of ALK-rearranged tumors (30, 32). In spite of this, the performance of the radiological model for predicting ALK status was not significantly enhanced with the addition of these conventional CT features. This phenomenon may be attributed to the inclusion of the LoG-processed features in our model. The LoG is a spatial filtering technique that enhances the marginal features from surrounding regions, which provides more information concerning tumour's surroundings. Dou et al.'s study revealed that radiomic features extracted from rims of tumors were able to predict distant metastases in locally advanced NSCLC (Concordance Index = 0.64) (33), which suggests that radiomic features can reflect the invasiveness of the tumors. In fact, radiomic features and conventional CT features were highly correlated. Stephen et al.'s study illustrated that one radiologist-defined imaging feature was associated with multiple radiomic features (21). In other words, radiomic features were expansions of the conventional CT features in detail to some degree. The finding in Stephen et al.'s study also explains another result that our radiological model had a much fewer number of features compared to the radiomic one at the final selection step.

In addition to the conventional CT features discussed above, we identified the intra-tumoural cavity and left lower lobe location were associated with the ALK mutation status. Previous studies found no difference in the prevalence of cavity between the ALK-mutated group and the control group, yet they either excluded both EGFR and ALK mutations in the ALK-negative group (12, 29, 34) or generalized the definition of cavity by including bubble lucence (12, 31). The lobar location preference for ALK mutations was only mentioned in Yoon's study (20). More studies are warranted to establish a tight connection between these two features and ALK mutations status in lung adenocarcinomas.

The integrated model contained radiomic, conventional CT and clinical features, and showed the highest AUC score (0.88) in both the primary and the test cohorts. The enhancement was statistically significant in the primary cohort but not in the test cohort. We found that the standard errors of the discriminative scores for patients with different ALK mutation statuses in the test cohort were higher than those in the primary cohort in the corresponding mutation group. It was also reflected by a wider range of confidence interval for AUC in the test cohort. The relatively large variance of discriminative scores for patients was partly due to the limited sample size in the test cohort. In spite of this, the improved efficacy of the integrated model by adding clinical characteristics for lesions in the primary cohort suggests that clinical information was effective to improve the radiomic-based model for detecting ALK-mutated status. Adding more ALK-associated clinical variables such as carcinoembryonic antigen (CEA) level and histological growth pattern may further enhance the performance of the model (35, 36). Previously, the best predictive model for the detection of ALK mutations was from Yamamoto's study (AUC = 0.846), in which it contained age as the only selected clinical feature and several conventional CT features (14). However, their work was based on enhanced CT images. The promising performance of the radiomic model in our study indicates that radiomic features extracted from non-enhanced CT images are adequate for establishing a convincing predictive model for ALK mutations in lung adenocarcinomas.

For the identified clinical features in our integrated model, smoking history had the highest discriminatory power (high weighting coefficient in both DT and LR), which is consistent with previous studies that observed more non-smokers in the ALK+ population (29, 30). Nonetheless, some integrated models for predicting ALK mutations did not remain smoking status as a significant index after their selection procedures (14, 20). This discrepancy may be caused by different model construction strategies and smoking cultures. Furthermore, we identified clinical stage I as an important clinical feature that was inversely associated with ALK rearrangements. This coincides with the finding that ALK mutations were more common in lung adenocarcinoma of stages III and IV in the univariate analysis. Similar results were found in Choi et al.'s study, in which ALK gene fusion was more likely to occur in lung cancer with a more advanced stage (37). We also noticed that the only shape-based radiomic feature—Major_Axis_Length was picked in the integrated model. It measures the largest axis length in a three-dimensional VOI. Most early studies measured the maximal diameter of tumors on a 2D plane and did not find a correlation between tumor size and ALK mutation (20, 29, 35, 38), while others found smaller diameters in ALK mutated tumors (39). Our study yielded a contradictory result that ALK-mutated tumors had a significantly larger diameter. These findings altogether suggest that the measurement of maximum diameter on a 2D plane is not representative of the real size of the tumor. Future studies should use the 3D axis length of tumors when building prediction models for better accuracy.

However, there are several limitations in our study. First, it is a retrospective study with patients from a single medical center. In the current study, we repeated the 10-fold cross-validation process 10 times to avoid overfitting and to minimize the optimism bias. Furthermore, an independent test cohort was used to validate the performance of our models. Despite, our model's generalizability should be further examined on data from a different medical center in the future. Second, we did not evaluate the effects of CEA and the maximum SUV value from PET/CT examination because such data were missing in approximately one-third of the patients. Third, we only examined radiomic and conventional features from the non-contrast enhanced CT images in this study due to the retrospective nature of the study. We can perform a prospective study to include features based on contrast-enhanced CT data of dual-energy scanning mode using dual-energy CT scanners to explore whether this can further improve the effectiveness of the predictive model in the future.

In conclusion, our findings highlight the feasibility of non-invasively predicting the ALK genetic status in lung adenocarcinomas using an integrated model that combines clinical, conventional CT, and radiomic features.

## Data Availability Statement

All datasets generated for this study are included in the article/Supplementary Material.

## Ethics Statement

The studies involving human participants were reviewed and approved by Ethics Review Committee of Peking Union Medical College Hospital, Chinese Academy of Medical Sciences. Written informed consent for participation was not required for this study in accordance with the national legislation and the institutional requirements.

## Author Contributions

LS, ZJ, and WS: study conceive and design. LS and ZZ: literature research. LS, ZZ, HW, and HD: data acquisition. LS, ZZ, LM, XL, and WH: data analysis and interpretation. LS and HD: evaluation the conventional thin-slice CT images. LS and ZZ: manuscript drafting. All authors manuscript revision for important intellectual content, approval of final version of submitted manuscript, manuscript editing, and had full access to all of the data in the study and take responsibility for the integrity of the data and the accuracy of the data analysis.

### Conflict of Interest

LM and XL were employed by the company Deepwise Inc. The remaining authors declare that the research was conducted in the absence of any commercial or financial relationships that could be construed as a potential conflict of interest.

## Abbreviations

ROC: receiver operating characteristic
AUC: area under the curve
CT: computed tomography
DICOM: digital imaging and communications in medicine
GGO: ground-glass opacity
GLCM: gray level co-occurrence matrix
GLSZM: gray level size zone matrix
GLRLM: gray level run-length matrix
GLDM: gray level dependence matrix
AIS: adenocarcinoma *in situ*
MIA: minimally invasive adenocarcinoma
IAC: invasive adenocarcinoma
NCCN: National Comprehensive Cancer Network
NSCLC: non-small cell lung cancer
CEA: carcinoembryonic antigen
DBSCAN: density-based spatial clustering of applications with noise
RFE: recursive feature elimination
LR: logistic regression
DT: decision tree
