# Supplementary material for: Clinical, Conventional CT and Radiomic Feature-Based Machine Learning Models for Predicting ALK Rearrangement Status in Lung Adenocarcinoma Patients
Source: Front Oncol. 2020 Mar 20;10:369. doi: 10.3389/fonc.2020.00369 (PMC7099003; doi:10.3389/fonc.2020.00369)
Supplement: Supplementary file 1 [file Data_Sheet_1.docx]

## *Supplementary Material*

1. **Supplementary Information**
   1. **Lesion segmentation**

The anonymized thin-slice images of non-enhanced CT in DICOM format were automatically delineated with automatic pulmonary nodule segmentation algorithms proposed by Qi et al (1). The detection model was a two-stage network that integrated both image and feature pyramids for nodule detection. The segmentation model was built based on the Recurrent Convolutional Neural Networks (RCNN), and the attention map was used to improve model performance. The multiplication of the original image patches and previous attention maps were fed into the network at each RCNN iteration. Both the detection model and segmentation model were trained on a combination of public and in-house datasets. To improve the segmentation performance, the model was pre-trained on the Lung Image Database Consortium (LIDC) and Image Database Resource Initiative (IDRI) datasets (910 nodules) and then fine-tuned on the in-house dataset (1748 nodules).

The repeatability of automatic segmentation has been validated previously (1). Fifteen patients underwent two CT scans at a short time interval, with the conventional dose increased by 1/5 to 1/4. After the automatic segmentation was performed, the volume of the lung nodules was calculated. The 95% confidence interval (CI) for the limits of agreement was achieved using the Bland-Altman analysis. The variability test revealed that the 95% CI for the limits of agreement ranged from -5.34% to 13.08% for the volume measurements, which was clinically acceptable.

**1.2 Pre-processing methods**

In this study, two filtering processes were performed to give additional first-order and texture characteristics. For the wavelet filter, a discrete, one-level and “Coiflet 1” three dimensional wavelet transformation was applied to each CT image, which decomposed the original image X into 8 decompositions. Consider H and L to be a high-pass function and low-pass function respectively, and the decomposition of X can be labeled as $X_{LLL}$, $X_{LLH}$,$X_{LHL}$,$X_{LHH}$,$X_{HLL}$,$X_{HLH}$, $X_{HHL}$ and$X_{HHH}$. The subscripts denoted the type of filters that were applied along x, y and z direction. The workflow is illustrated in **Figure S1.2.1**.


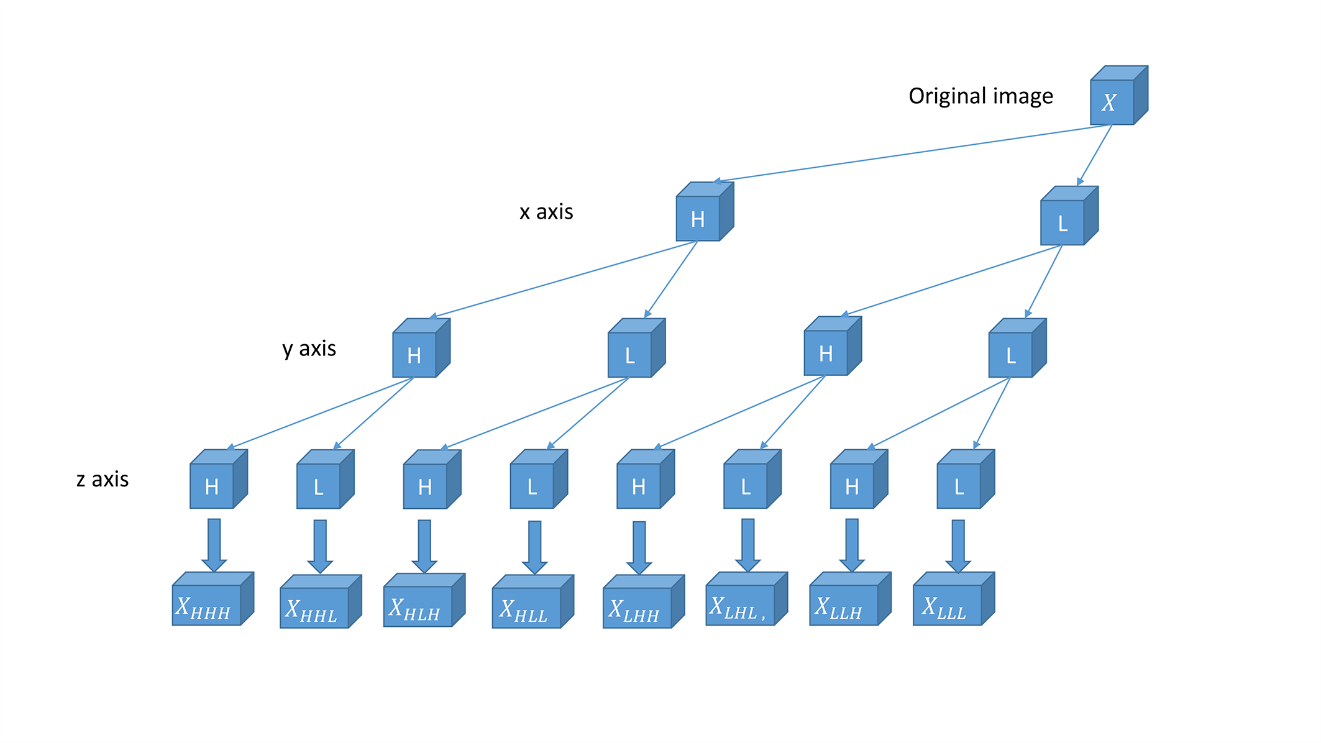


**Figure S1.2.1.** Schematic of the three dimensional wavelet transform applied to each CT image. The original image $X$ is decomposed into 8 decompositions, by directional low-pass and high-pass filtering: $X_{LLL}$, $X_{LLH}$,$X_{LHL}$,$X_{LHH}$,$X_{HLL}$,$X_{HLH}$, $X_{HHL}$ and$X_{HHH}$.

The Laplacian of Gaussian (LoG) filter was consisted of a proceeding Gaussian filter and a Laplacian filter. The Gaussian kernel is used to smooth the image and reduce noise and the Laplacian operator is sensitive to areas with rapidly changing intensities. The LoG was often used to intensify the edge information of interested area. The Gaussian kernel is defined as

$$G\left( x,y,z,\sigma\right)= \frac{1}{{(\sigma\sqrt{2\pi})}^{3}}e^{-\frac{x^{2}+y^{2}+z^{2}}{2\sigma^{2}}}$$

which is then convolved by the Laplacian kernel $\nabla^{2}G(x,y,z)$. The width of the filter in the Gaussian kernel is determined by σ and was set at 1, 2, 3, 4 and 5 in this study. Lower value is associated with more fine texture and higher value is associated with more coarse textures.

**1.3 Radiomic features**

Radiomic features were divided into three groups according to different types: (1) first-order features (describing the image intensity); (2) shape features (describing the 2D and 3D morphology of the tumour); (3) texture features (describing the interior and surface texture of the tumour). And texture features included grey level co-occurrence matrix (GLCM) features, grey level size zone matrix (GLSZM) features, grey level run-length matrix (GLRLM) features and grey level dependency matrix (GLDM) features.

1. First-order features, also known as histogram features, include 18 features, which describe the distribution of voxel intensities within the volume of interest (VOI).
2. Shape features include 4 features, which describe the morphological property of the tumour’s outline.
3. GLCM features include 22 features, which describe the spatial dependence of each pixel in the tumour region. (N3 = 15) (2).
4. GLSZM features include 16 features, which quantify the grey level zone in the tumour region (3). It is an advanced statistical matrix for textural feature characterization.
5. GLRLM features include 16 features, which quantify grey level runs and describe the textural characteristics of the tumour region (4).
6. GLDM features include 14 features, which describe the complex textural characteristics in the tumour region. This algorithm is insensitive to monotonic grey level transformation (5).

First-order features and texture features were obtained from 14 types of images that were composed of one original type, eight wavelet-filtered types and five LoG-filtered types. Shape features were only obtained from the original images for that filtered images no longer reflect the true morphology of tumours. For each VOI, a total of 1218 quantitative imaging features were extracted. Among them, there were 252 (18 types of features $\times$14 types of images) first-order features, 14 shape features, 308 (22 types of features $\times$14 types of images) GLCM features, 224 (16 types of features $\times$14 types of images) GLSZM features, 224 (16 types of features $\times$14 types of images) GLRLM features and 196 (14 types of features $\times$14 types of images) GLDM features. The features in each group were listed below. For formulas of each feature, please refer to the PyRadiomics official website: <https://pyradiomics.readthedocs.io/en/latest/features.html>

***Group 1. First-order features***

First-order statistics describe the distribution of voxel intensities within the CT image region defined by the mask through commonly used and basic metrics. X is a set of Np voxels included in the VOI.

**Table S1.3.1.** Descriptions of all features for first-order characteristics.

| **No.** | **Feature names** | **Descriptions** |
| --- | --- | --- |
| 1 | 10 Percentile | The 10th percentile of X |
| 2 | 90 Percentile | The 90th percentile of X |
| 3 | Energy | Energy is a measure of the magnitude of voxel values in an image. A larger values implies a greater sum of the squares of these values. |
| 4 | Entropy | Entropy specifies the uncertainty/randomness in the image values. It measures the average amount of information required to encode the image values. |
| 5 | Interquartile Range | Here P25 and P75 are the 25^th^ and 75^th^ percentile of the image array, respectively. |
| 6 | Kurtosis | Kurtosis is a measure of the ‘peakedness’ of the distribution of values in the image VOI. A higher kurtosis implies that the mass of the distribution is concentrated towards the tail(s) rather than towards the mean. A lower kurtosis implies the reverse: that the mass of the distribution is concentrated towards a spike near the Mean value. |
| 7 | Maximum | The maximum grey level intensity within the VOI. |
| 8 | Mean Absolute Deviation | Mean Absolute Deviation is the mean distance of all intensity values from the Mean Value of the image array. |
| 9 | Mean | The average grey level intensity within the VOI. |
| 10 | Median | The median grey level intensity within the VOI. |
| 11 | Minimum | The minimum grey level intensity within the VOI. |
| 12 | Range | The range of grey values in the VOI. |
| 13 | Robust Mean Absolute Deviation | Robust Mean Absolute Deviation is the mean distance of all intensity values from the Mean Value calculated on the subset of image array with grey levels in between, or equal to the 10th and 90th percentile. |
| 14 | Root Mean Squared (RMS) | RMS is the square-root of the mean of all the squared intensity values. It is another measure of the magnitude of the image values. |
| 15 | Skewness | Skewness measures the asymmetry of the distribution of values about the Mean value. Depending on where the tail is elongated and the mass of the distribution is concentrated, this value can be positive or negative. |
| 16 | Total Energy | Total Energy is the value of Energy feature scaled by the volume of the voxel in cubic mm. |
| 17 | Uniformity | Uniformity is a measure of the sum of the squares of each intensity value. This is a measure of the homogeneity of the image array, where a greater uniformity implies a greater homogeneity or a smaller range of discrete intensity values. |
| 18 | Variance | Variance is the mean of the squared distances of each intensity value from the Mean value. This is a measure of the spread of the distribution about the mean. |

***Group 2. Shape features***

Shape features describe the morphological property of the VOI and were generated from only the image without filtration. $N_{v}$ represents the number of voxels included in the VOI. $N_{f}$ represents the number of faces (triangles) defining the Mesh.

**Table S1.3.2.** Descriptions of all features for shape-based characteristics.

| **No.** | **Feature names** | **Descriptions** |
| --- | --- | --- |
| 1 | Elongation | Elongation shows the relationship between the two largest principal components in the VOI shape. |
| 2 | Flatness | Flatness shows the relationship between the largest and smallest principal components in the VOI shape. |
| 3 | Least Axis Length | This feature yields the smallest axis length of the VOI-enclosing ellipsoid and is calculated using the largest principal component  $\lambda_{\mathrm{least}}$. |
| 4 | Major Axis Length | This feature yields the largest axis length of the VOI-enclosing ellipsoid and is calculated using the largest principal component  $\lambda_{major}$. |
| 5 | Maximum 2D Diameter Column | Maximum 2D diameter (column) is defined as the largest pairwise Euclidean distance between tumour surface mesh vertices in the row-slice (usually the coronal) plane. |
| 6 | Maximum 2D Diameter Row | Maximum 2D diameter (row) is defined as the largest pairwise Euclidean distance between tumour surface mesh vertices in the column-slice (usually the sagittal) plane. |
| 7 | Maximum 2D Diameter Slice | Maximum 2D diameter (slice) is defined as the largest pairwise Euclidean distance between tumour surface mesh vertices in the row-column (generally the axial) plane. |
| 8 | Maximum 3D Diameter | Maximum 3D diameter is defined as the largest pairwise Euclidean distance between tumour surface mesh vertices. |
| 9 | Mesh Volume | The volume of the VOI (V) is calculated from the triangle mesh of the VOI. For each face ii in the mesh, defined by points $a_{i}$,$b_{i}$, and $c_{i}$, the (signed) volume $V_{f}$ of the tetrahedron defined by that face and the origin of the image (O) is calculated. The sign of the volume is determined by the sign of the normal, which must be consistently defined as either facing outward or inward of the VOI. |
| 10 | Minor Axis Length | This feature yields the second-largest axis length of the VOI-enclosing ellipsoid and is calculated using the largest principal component $\lambda_{\mathrm{minor}}$. |
| 11 | Sphericity | Sphericity is a measure of the roundness of the shape of the tumour region relative to a sphere. It is a dimensionless measure, independent of scale and orientation. The value range is 0$<$sphericity$\leq$1, where a value of 1 indicates a perfect sphere (a sphere has the smallest possible surface area for a given volume, compared to other solids). |
| 12 | Surface Area | To calculate the surface area, first the surface area Ai of each triangle in the mesh is calculated. The total surface area is then obtained by taking the sum of all calculated sub-areas. |
| 13 | Surface Volume Ratio | A lower value indicates a more compact (sphere-like) shape. This feature is not dimensionless, and is therefore (partly) dependent on the volume of the VOI. |
| 14 | Voxel Volume | The volume of the VOI is approximated by multiplying the number of voxels in the VOI by the volume of a single voxel. This is a less precise approximation of the volume and is not used in subsequent features. This feature does not make use of the mesh and is not used in calculation of other shape features. |

***Group 3. Grey-Level Co-Occurrence Matrix (GLCM) features***

A GLCM is defined as $P(i,j;\delta,\alpha)$, a matrix with size $N_{g}\times N_{g}$ describing the second-order joint probability function of an image, where the $(i,j)$^th^ element represents the number of times the combination of intensity levels $i$ and $j$ occur in two pixels in the image, that are separated by a distance of $\delta$ pixels in direction $\alpha$, and $N_{g}$ is the number of discrete grey level intensities. In this study, distance $\delta$ was set to 1 and direction $\alpha$ to each of the 13 directions in three dimensions, yielding a total of 13 grey level co-occurrence matrices for each 3D image. From these grey-level co-occurrence matrices, several textural features are derived. Each 3D grey level co-occurrence based feature was then calculated as the mean of the feature calculations for each of the 13 directions.

**Table S1.3.3.** Descriptions of all features for GLCM characteristics.

| **No.** | **Feature names** | **Descriptions** |
| --- | --- | --- |
| 1 | Auto Correlation | Auto correlation is a measure of the magnitude of the fineness and coarseness of texture. |
| 2 | Joint Average | The mean grey level intensity of the $i$ distribution. |
| 3 | Cluster Prominence | Cluster Prominence is a measure of the skewness and asymmetry of the GLCM. A higher values implies more asymmetry about the mean while a lower value indicates a peak near the mean value and less variation about the mean. |
| 4 | Cluster Shade | Cluster Shade is a measure of the skewness and uniformity of the GLCM. A higher cluster shade implies greater asymmetry about the mean. |
| 5 | Cluster Tendency | Cluster Tendency is a measure of groupings of voxels with similar grey-level values. |
| 6 | Contrast | Contrast is a measure of the local intensity variation, favouring values away from the diagonal (i=j). A larger value correlates with a greater disparity in intensity values among neighboring voxels. |
| 7 | Correlation | Correlation is a value between 0 (uncorrelated) and 1 (perfectly correlated) showing the linear dependency of grey level values to their respective voxels in the GLCM. |
| 8 | Difference Average | Difference Average measures the relationship between occurrences of pairs with similar intensity values and occurrences of pairs with differing intensity values. |
| 9 | Difference Entropy | Difference Entropy is a measure of the randomness/variability in neighborhood intensity value differences. |
| 10 | Difference Variance | Difference Variance is a measure of heterogeneity that places higher weights on differing intensity level pairs that deviate more from the mean. |
| 11 | Joint Energy | Energy is a measure of homogeneous patterns in the image. A greater Energy implies that there are more instances of intensity value pairs in the image that neighbor each other at higher frequencies. |
| 12 | Joint Entropy | Joint entropy is a measure of the randomness/variability in neighborhood intensity values. |
| 13 | Informational Measure of Correlation 1 (IMC1) | IMC1 assesses the correlation between the probability distributions of i and j, quantifying the complexity of the texture. |
| 14 | Informational Measure of Correlation 2(IMC2) | IMC2 also assesses the correlation between the probability distributions of i and j, quantifying the complexity of the texture. |
| 15 | Inverse Difference Moment (IDM) | IDM (also known as Homogeneity 2) is a measure of the local homogeneity of an image. |
| 16 | Inverse Difference Moment Normalized (IDMN) | IDMN is a measure of the local homogeneity of an image. |
| 17 | Inverse Difference (ID) | ID (also known as Homogeneity 1) is another measure of the local homogeneity of an image. With more uniform grey levels, the denominator will remain low, resulting in a higher overall value. |
| 18 | Inverse Difference Normalized (IDN) | IDN is another measure of the local homogeneity of an image. Unlike Homogeneity 1, IDN normalizes the difference between the neighboring intensity values by dividing over the total number of discrete intensity values. |
| 19 | Inverse Variance | Inverse-variance weighting is a method of aggregating two or more random variables to minimize the variance of the weighted average. |
| 20 | Maximum Probability | Maximum Probability is occurrences of the most predominant pair of neighboring intensity values. |
| 21 | Sum Entropy | The sum of neighborhood intensity value differences. |
| 22 | Sum Squares | A measure in the distribution of neighboring intensity level pairs about the mean intensity level in the GLCM. |

***Group 4. Grey-Level Size Zone Matrix (GLSZM) features***

A grey level size zone matrix (GLSZM) quantifies grey level zones in an image. A grey level zone is defined as the number of connected voxels that share the same grey level intensity. A voxel is considered connected if the distance is 1 according to the infinity norm (26-connected region in a 3D, 8-connected region in 2D). In a grey level size zone matrix $P(i,j)$ the ${(i,j)}^{th}$ element equals the number of zones with grey level i and size j appear in image. Contrary to GLCM and GLRLM, the GLSZM is rotation independent, with only one matrix calculated for all directions in the VOI.

**Table S1.3.4.** Descriptions of all features for GLSZM characteristics.

| **No.** | **Feature names** | **Descriptions** |
| --- | --- | --- |
| 1 | Grey Level Non-Uniformity (GLN) | GLN measures the variability of grey-level intensity values in the image, with a lower value indicating more homogeneity in intensity values. |
| 2 | Grey Level Non-Uniformity Normalized (GLNN) | GLNN measures the variability of grey-level intensity values in the image, with a lower value indicating a greater similarity in intensity values. This is the normalized version of the GLN formula. |
| 3 | Grey Level Variance (GLV) | GLV measures the variance in grey level intensities for the zones. |
| 4 | High Grey Level Zone Emphasis (HGLZE) | HGLZE measures the distribution of the higher grey-level values, with a higher value indicating a greater proportion of higher grey-level values and size zones in the image. |
| 5 | Large Area Emphasis (LAE) | LAE is a measure of the distribution of large area size zones, with a greater value indicative of larger size zones and more coarse textures. |
| 6 | Large Area High Grey Level Emphasis (LAHGLE) | LAHGLE measures the proportion in the image of the joint distribution of larger size zones with higher grey-level values. |
| 7 | Large Area Low Grey Level Emphasis (LALGLE) | LALGLE measures the proportion in the image of the joint distribution of larger size zones with lower grey-level values. |
| 8 | Low Grey Level Zone Emphasis (LGLZE) | LGLZE measures the distribution of lower grey-level size zones, with a higher value indicating a greater proportion of lower grey-level values and size zones in the image. |
| 9 | Size Zone Non-Uniformity (SZN) | SZN measures the variability of size zone volumes in the image, with a lower value indicating more homogeneity in size zone volumes. |
| 10 | Size Zone Non-Uniformity Normalized (SZNN) | SZNN measures the variability of size zone volumes throughout the image, with a lower value indicating more homogeneity among zone size volumes in the image. This is the normalized version of the SZN formula. |
| 11 | Small Area Emphasis (SAE) | SAE is a measure of the distribution of small size zones, with a greater value indicative of smaller size zones and more fine textures. |
| 12 | Small Area High Grey Level Emphasis (SAHGLE) | SAHGLE measures the proportion in the image of the joint distribution of smaller size zones with higher grey-level values. |
| 13 | Small Area Low Grey Level Emphasis (SALGLE) | SALGLE measures the proportion in the image of the joint distribution of smaller size zones with lower grey-level values. |
| 14 | Zone Entropy | Measures the uncertainty/randomness in the distribution of zone sizes and grey levels. A higher value indicates more heterogeneity in the texture patterns. |
| 15 | Zone Percentage | Measures the coarseness of the texture by taking the ratio of number of zones and number of voxels in the VOI. |
| 16 | Zone Variance | Measures the variance in zone size volumes for the zones. |

***Group 5. Grey-Level Run-Length Matrix (GLRLM) features***

Run length metrics quantify grey level runs in an image. A grey level run is defined as the length in number of pixels, of consecutive pixels that have the same grey level value. In a grey level run length matrix $p(i,j|\theta)$, the $(i,j)$th element describes the number of times $j$ a grey level $i$ appears consecutively in the direction specified by $\theta$, and $N_{g}$ is the number of discrete grey level intensities. In this study, a GLRL matrix was computed for every of the 13 directions in three dimensions, from which the below textural features were derived. Each 3D GLRLM feature was then calculated as the mean of the feature values for each of the 13 directions.

**Table S1.3.5.** Descriptions of all features for GLRLM characteristics.

| **No.** | **Feature names** | **Descriptions** |
| --- | --- | --- |
| 1 | Grey Level Non Uniformity (GLN) | GLN measures the similarity of grey-level intensity values in the image, where a lower GLN value correlates with a greater similarity in intensity values. |
| 2 | Grey Level NonUniformity Normalized (GLNN) | GLNN measures the similarity of grey-level intensity values in the image, where a lower GLNN value correlates with a greater similarity in intensity values. This is the normalized version of the GLN formula. |
| 3 | Grey Level Variance (GLV) | GLV measures the variance in grey level intensity for the runs. |
| 4 | High Grey Level Run Emphasis (HGLRE) | HGLRE measures the distribution of the higher grey-level values, with a higher value indicating a greater concentration of high grey-level values in the image. |
| 5 | Long Run Emphasis (LRE) | LRE is a measure of the distribution of long run lengths, with a greater value indicative of longer run lengths and more coarse structural textures. |
| 6 | Long Run High Grey Level Emphasis (LRHGLE) | LRHGLE measures the joint distribution of long run lengths with higher grey-level values. |
| 7 | Long Run Low Grey Level Emphasis (LRLGLE) | LRLGLE measures the joint distribution of long run lengths with lower grey-level values. |
| 8 | Low Grey Level Run Emphasis (LGLRE) | LGLRE measures the distribution of low grey-level values, with a higher value indicating a greater concentration of low grey-level values in the image. |
| 9 | Run Entropy (RE) | RE measures the uncertainty/randomness in the distribution of run lengths and grey levels. A higher value indicates more heterogeneity in the texture patterns. |
| 10 | Run Length Non-Uniformity (RLN) | RLN measures the similarity of run lengths throughout the image, with a lower value indicating more homogeneity among run lengths in the image. |
| 11 | Run Length Non-Uniformity Normalized (RLNN) | RLNN measures the similarity of run lengths throughout the image, with a lower value indicating more homogeneity among run lengths in the image. This is the normalized version of the RLN formula. |
| 12 | Run Percentage (RP) | RP measures the coarseness of the texture by taking the ratio of number of runs and number of voxels in the VOI. |
| 13 | Run Variance | A measure of the variance in runs for the run lengths. |
| 14 | Short Run Emphasis (SRE) | SRE is a measure of the distribution of short run lengths, with a greater value indicative of shorter run lengths and more fine textural textures. |
| 15 | Short Run High Grey Level Emphasis (SRHGLE) | SRHGLE measures the joint distribution of shorter run lengths with higher grey-level values. |
| 16 | Short Run Low Grey Level Emphasis (SRLGLE) | SRLGLE measures the joint distribution of shorter run lengths with lower grey-level values. |

***Group 6. Grey-Level Dependence Matrix (GLDM) features***

A grey level dependence matrix (GLDM) quantifies grey level dependencies in an image. A grey level dependency is defined as a the number of connected voxels within distance δ that are dependent on the center voxel. A neighbouring voxel with grey level $j$ is considered dependent on center voxel with grey level $i$ if $\left| i-j \right|\leq\alpha$. In a grey level dependence matrix $P(i,j)$ the ${(i,j)}^{th}$element describes the number of times a voxel with grey level $i$ with $j$ dependent voxels in its neighbourhood appears in image.

**Table S1.3.6.** Descriptions of all features for GLDM characteristics.

| **No.** | **Feature names** | **Descriptions** |
| --- | --- | --- |
| 1 | Dependence Entropy | A measure of the randomness/variability in dependence size in the image. |
| 2 | Dependence Non-Uniformity (DN) | DN measures the similarity of dependence throughout the image, with a lower value indicating more homogeneity among dependencies in the image. |
| 3 | Dependence Non-Uniformity Normalized (DNN) | DNN measures the similarity of dependence throughout the image, with a lower value indicating more homogeneity among dependencies in the image. This is the normalized version of the DN formula. |
| 4 | Dependence Variance (DV) | DV measures the variance in dependence size in the image. |
| 5 | Grey Level Non-Uniformity (GLN) | GLN measures the similarity of grey-level intensity values in the image, where a lower GLN value correlates with a greater similarity in intensity values. |
| 6 | Grey Level Variance (GLV) | GLV measures the variance in grey level in the image. |
| 7 | High Grey Level Emphasis (HGLE) | HGLE measures the distribution of the higher grey-level values, with a higher value indicating a greater concentration of high grey-level values in the image. |
| 8 | Large Dependence Emphasis (LDE) | A measure of the distribution of large dependencies, with a greater value indicative of larger dependence and more homogeneous textures. |
| 9 | Large Dependence High Grey Level Emphasis (LDHGLE) | LDHGLE measures the joint distribution of large dependence with higher grey-level values. |
| 10 | Large Dependence Low Grey Level Emphasis (LDLGLE) | LDLGLE measures the joint distribution of large dependence with lower grey-level values. |
| 11 | Low Grey Level Emphasis (LGLE) | LGLE measures the distribution of low grey-level values, with a higher value indicating a greater concentration of low grey-level values in the image. |
| 12 | Small Dependence Emphasis (SDE) | A measure of the distribution of small dependencies, with a greater value indicative of smaller dependence and less homogeneous textures. |
| 13 | Small Dependence High Grey Level Emphasis (SDHGLE) | SDHGLE measures the joint distribution of small dependence with higher grey-level values. |
| 14 | Small Dependence Low Grey Level Emphasis (SDLGLE) | SDLGLE measures the joint distribution of small dependence with lower grey-level values. |

**References**

1. Qi LL, Wu BT, Tang W, Zhou LN, Huang Y, Zhao SJ, et al. Long-term follow-up of persistent pulmonary pure ground-glass nodules with deep learning-assisted nodule segmentation. *Eur Radiol* (2019). Epub 2019/09/06. doi: 10.1007/s00330-019-06344-z. PubMed PMID: 31485837.

2. Haralick R, Shanmugam K, Dinstein I. Textural Features for Image Classification. *IEEE Transactopns on Systems, Man, and Cybernetics* (1973) SMC-3(6):610-21. doi: 10.1109/TSMC.1973.4309314.

3. Thibault G, Fertil B, Navarro C, Pereira S, Cau P, Levy N, et al., editors. Texture Indexes and Gray Level Size Zone Matrix Application to Cell Nuclei Classification. *10th International Conference on Pattern Recognition and Information Processing*; 2009; Minsk, Belarus.

4. Tang X. Texture Information in Run-Length Matrices. *IEEE Transactions on Image Processing* (1998) 7(11):1602-9. doi: 10.1109/83.725367.

5. Sun C, Wee W. Neighboring Gray Level Dependence Matrix for Texture Classification. *Computer Vision, Graphics, and Image Processing* (1982) 23:341-52. doi: <https://doi.org/10.1016/0734-189X(83)90032-4>.
