# Supplementary material for: Clinical, Conventional CT and Radiomic Feature-Based Machine Learning Models for Predicting ALK Rearrangement Status in Lung Adenocarcinoma Patients
Source: Front Oncol. 2020 Mar 20;10:369. doi: 10.3389/fonc.2020.00369 (PMC7099003; doi:10.3389/fonc.2020.00369)
Supplement: Supplementary file 2 [file Table_1.docx]

## *Supplementary Material*

1. **Supplementary Tables**

**Supplementary Table 1.** The definitions and scoring rules for clinical characteristics and conventional CT features

| **Feature** | **Definition** | **Scoring** |  |
| --- | --- | --- | --- |
| **Clinical features** | | |  |
| \| Age \| Age of patients \| Unit, years old \| \| --- \| --- \| --- \| \| Sex \| Gender of patients \| 1, male; 2, female \| \| Smoking history \| The history of smoking cigarettes \| 0, non-smoker; 1-current smoker; 2, former smoker \| \| Smoking status (1) \| Smoking status was divided based on smoking index (SI, unit, pack-year), SI = number of packs per day $\times$ number of years of smoking \| 1, light smoker (SI ≤ 10); 2, moderate smoker (10 < SI < 20); 3, heavy smoker (SI ≥ 20) \| | | |  |
| \| Clinical stage \| The clinical TNM stage was determined according to the 8th edition of the American Cancer Society guideline for non-small-cell lung cancer staging \| 1, stage I; 2, stage II; 3, stage III; 4, stage IV \| \| --- \| --- \| --- \| | | |  |
| \| Distal metastasis status \| Distant metastases including intracranial, hepatic, bone, or other distant organs metastases were diagnosed through contrast-enhanced head MR, contrast-enhanced abdomen CT or liver ultrasound, whole-body bone scan or ^18^F-FDG-PET/CT. \| 0, no; 1, yes \| \| --- \| --- \| --- \| | | |  |
| \| Tumour invasiveness \| The pathological subtypes of adenocarcinoma were assessed based on tumour’s invasiveness according to the latest International Multidisciplinary Classification of lung adenocarcinoma \| 1, adenocarcinoma in situ (AIS); 2, minimally invasive adenocarcinoma (MIA); 3, invasive adenocarcinoma (IAC) \| \| --- \| --- \| --- \| | | |  |
| **Conventional CT features** |  |  |  |
| Maximum diameter | Longest diameter of the maximum cross section | Unit, millimeter |  |
| Mean CT value | Mean CT value measured on the maximum cross section, avoiding vascular, cavity and necrosis during measurements | Unit, Hounsfield unit |  |
| Lesion location | Central, tumour originated from the segmental or more proximal bronchi; peripheral, tumour originated from the subsegmental bronchi or more distal airway | 1, central; 2, peripheral |  |
| Lobe of lesion located | LLL, left lower lobe; LUL, left upper lobe; RLL, right lower lobe; RML, right middle lobe; RUL, right upper lobe; mixed, lesions across lobes | 1, LLL; 2, LUL; 3, RLL; 4, RML; 5, RUL; 6, mixed |  |
| Margin | Spiculated, short lines radiating from tumour margin；irregular, irregularity of tumour margin; lobulated, surface of the tumour that showed a wavy or scalloped configuration; smooth, no spiculation or lobulation or irregularity of tumour margin | 1, spiculated or irregular;2, lobulated; 3, smooth |  |
| Density | Pure ground-glass opacity, unmasking the bronchi and vascular branches below; partial solid, presence of ground-glass opacity and solid components; solid, absence of ground-glass opacity | 1, pure ground-glass opacity (GGO);2, partial solid; 3, solid |  |
| Local lymphadenopathy | Enlarged local lymph nodes (hilar or mediastinal) with  short-axis diameter greater than 1 cm | 0, no; 1, yes |  |
| ***Internal characteristics*** |  |  |  |
| Cavity | A larger cavity or space (>5mm) in the tumour because of intratumoural necrosis | 0, no; 1, yes |  |
| Calcification | Containing calcification within the tumour | 0, no; 1, yes |  |
| ***External characteristics*** | |  |  |
| Pleural retraction sign | Retraction of the pleura toward the tumour | 0, no; 1, yes |  |
| Pleural effusion | Effusion seen in the thorax | 0, no; 1, yes |  |
| Pericardial effusion | Effusion seen in the pericardial cavity | 0, no; 1, yes |  |

**Supplementary Table 2.** Hyper-parameters for each selection method and classifier in the radiomic, the radiological and the integrated model.

| Models | Selection method (hyper-parameter names) | | |  | Classifier | |
| --- | --- | --- | --- | --- | --- | --- |
|  | F-test based (feature number) | DBSCAN  (eps, min sample number) | RFE (feature number) |  | LR (penalty) | DT (min impurity decrease) |
| Radiomic | 250 | 0.15, 3 | 30 |  | 1 | 0.0001 |
| Radiological | 350 | 0.2, 2 | 20 |  | 1 | 0.001 |
| Integrated | 350 | 0.25, 1 | 30 |  | 1 | 0.0001 |

**Supplementary Table 3.** Selected features and their weight coefficients in the Decision Tree (DT) and the Logistic Regression (LR) classifier in the radiomic model.

| **Feature types** | **Feature names** | **DT** | **LR** |
| --- | --- | --- | --- |
| **First-order (12)** | Original_Firstorder_90Percentile | 0.25 | 3.76 |
|  | Original_Firstorder_Entropy | 0.02 | -1.44 |
|  | Original_Firstorder_Maximum | 0.06 | 0.33 |
|  | Wavelet-LHH_Firstorder_10Percentile | 0.06 | 0 |
|  | Wavelet-HLL_Firstorder_Median | 0.03 | 0 |
|  | Wavelet-HHH_Firstorder_Mean | 0.03 | 0 |
|  | LoG-sigma-1-0-mm-3D_Firstorder_Median | 0.04 | -1.13 |
|  | LoG-sigma-1-0-mm-3D_Firstorder_RootMeanSquared | 0.07 | -1.26 |
|  | LoG-sigma-1-0-mm-3D_Firstorder_Minimum | 0 | 0 |
|  | LoG-sigma-2-0-mm-3D_Firstorder_10Percentile | 0.05 | 0 |
|  | LoG-sigma-3-0-mm-3D_Firstorder_90Percentile | 0.01 | 0 |
|  | LoG-sigma-5-0-mm-3D_Firstorder_Skewness | 0.06 | -0.50 |
| **GLCM (6)** | Original_ GLCM _ClusterShade | 0 | 0 |
|  | Wavelet-LHH_ GLCM _Correlation | 0 | -1.46 |
|  | Wavelet-LHL_GLCM_IDN | 0.06 | 0 |
|  | Wavelet-HHH_ GLCM _IMC1 | 0.02 | 0 |
|  | LoG-sigma-1-0-mm-3D_ GLCM _Autocorrelation | 0.05 | 0 |
|  | LoG-sigma-2-0-mm-3D_ GLCM _InverseVariance | 0 | 0.49 |
| **GLSZM (8)** | Original_GLSZM_SAHGLE | 0 | -0.17 |
|  | Wavelet-HHH_GLSZM_SALGLE | 0 | -0.78 |
|  | Wavelet-HLL_GLSZM_ZoneEntropy | 0.02 | 0 |
|  | Wavelet-HLH_GLSZM_ZoneEntropy | 0 | 0 |
|  | LoG-sigma-2-0-mm-3D_GLSZM_ZoneEntropy | 0 | -0.64 |
|  | LoG-sigma-3-0-mm-3D_GLSZM_SAE | 0.02 | 0 |
|  | LoG-sigma-3-0-mm-3D_GLSZM_SZNN | 0 | 1.11 |
|  | LoG-sigma-5-0-mm-3D_GLSZM_GLNN | 0.10 | 0 |
| **GLDM (2)** | Wavelet-LHH_GLDM_LDHGLE | 0.04 | 0.21 |
|  | LoG-sigma-1-0-mm-3D_GLDM_HGLE | 0 | 0 |
| **GLRLM (2)** | LoG-sigma-3-0-mm-3D_GLRLM_RunPercentage | 0 | 0 |
|  | LoG-sigma-4-0-mm-3D_ GLRLM _LRLGLE | 0.02 | 0 |

Note: The features were first listed based on their types. Within each type, the features were grouped by their associated pre-processing methods. The value of DT denotes the decrease of Gini index when such feature was selected in the DT model. A high DT value suggests more significant influence in differentiating ALK mutated status and non-ALK mutated status. It is non-directional. The value associated with the LR is the beta coefficient. Since all features were rescaled before entering selection procedure, these coefficients are equivalent to normalized LR coefficients. It is directional. A feature with a higher positive value suggests a stronger association with ALK-rearranged tumours.

**Supplementary Table 4.** Selected features and their weight coefficients in the Decision Tree (DT) and the Logistic Regression (LR) classifier in the radiological model.

| **Feature types** | **Feature names** | **DT** | **LR** |
| --- | --- | --- | --- |
| **Conventional CT (4)** | Pericardial effusion | 0 | 1.98 |
|  | Local lymphadenopathy | 0 | 1.05 |
|  | Lobulated margin | 0 | 0.80 |
|  | No pleural retraction sign | 0 | 0.57 |
| **First-order (11)** | Original_Firstorder_90Percentile | 0.13 | 3.11 |
|  | Original_Firstorder_InterquartileRange | 0.03 | -0.61 |
|  | Wavelet-LLL_Firstorder_Skewness | 0.22 | 0 |
|  | Wavelet-LHL_Firstorder_Mean | 0.08 | 0 |
|  | Wavelet-HLL_Firstorder_Mean | 0.06 | 0 |
|  | Wavelet-HHH_Firstorder_Kurtosis | 0.06 | 0.84 |
|  | Wavelet-LLL_Firstorder_Maximum | 0.01 | 0 |
|  | LoG-sigma-1-0-mm-3D_Firstorder_Mean | 0.09 | -3.42 |
|  | LoG-sigma-1-0-mm-3D_Firstorder_Minimum | 0 | 0 |
|  | LoG-sigma-3-0-mm-3D_Firstorder_Maximum | 0.09 | 0.38 |
|  | LoG-sigma-4-0-mm-3D_Firstorder_Skewness | 0.03 | -0.49 |
| **GLCM (4)** | Original_GLCM_Correlation | 0.01 | -1.69 |
|  | Original_GLCM_ClusterShade | 0.01 | -0.04 |
|  | LoG-sigma-4-0-mm-3D_GLCM_IMC2 | 0.08 | 0.45 |
|  | LoG-sigma-5-0-mm-3D_GLCM_InverseVariance | 0.03 | -0.04 |
| **GLSZM (1)** | Wavelet-HHH_GLSZM_SALGLE | 0.07 | -0.15 |

Note: The features were first listed based on their types. Within each type, the features were grouped by their associated pre-processing methods. The value of DT denotes the decrease of Gini index when such feature was selected in the DT model. A high DT value suggests more significant influence in differentiating ALK mutated status and non-ALK mutated status. It is non-directional. The value associated with the LR is the beta coefficient. Since all features were rescaled before entering selection procedure, these coefficients are equivalent to normalized LR coefficients. It is directional. A feature with a higher positive value suggests a stronger association with ALK-rearranged tumours.

**Supplementary Table 5.** Selected features and their weight coefficients in the Decision Tree (DT) and the Logistic Regression (LR) classifier in the integrated model.

| **Feature types** | **Feature names** | **DT** | **LR** |
| --- | --- | --- | --- |
| **Clinical (3)** | Current smoker | 0.11 | -1.67 |
|  | Stage I | 0 | -0.78 |
|  | Male | 0 | -0.15 |
| **Conventional CT (7)** | Local lymphadenopathy | 0 | 1.33 |
|  | Pericardial effusion | 0 | 1.14 |
|  | Left Lower Lobe (LLL) lesion | 0 | 0.67 |
|  | No cavity in the lesion | 0 | -0.58 |
|  | Lobulated margin | 0 | 0.52 |
|  | No pleural retraction sign | 0 | 0.34 |
|  | No local lymphadenopathy | 0.17 | 0 |
| **First-order (7)** | Wavelet-HHL_Firstorder_Kurtosis | 0.09 | 1.02 |
|  | Wavelet-HLL_Firstorder_Median | 0.06 | -0.96 |
|  | Wavelet-LHH_Firstorder_Skewness | 0.03 | 0.12 |
|  | Wavelet-LLL_Firstorder_Minimum | 0.04 | 0 |
|  | Wavelet-HLH_Firstorder_Median | 0.03 | 0 |
|  | LoG-sigma-1-0-mm-3D_Firstorder_Minimum | 0 | 0 |
|  | LoG-sigma-2-0-mm-3D_Firstorder_Minimum | 0.02 | 0 |
| **GLCM (7)** | Wavelet-LLL_GLCM_ClusterShade | 0.04 | -2.08 |
|  | Wavelet-LLH_GLCM_IMC2 | 0.03 | 0.35 |
|  | Wavelet-HLH_GLCM_IMC2 | 0.02 | 0 |
|  | Wavelet-HLH_GLCM_IMC1 | 0.01 | 0 |
|  | LoG-sigma-1-0-mm-3D_GLCM_IMC1 | 0.06 | 0 |
|  | LoG-sigma-3-0-mm-3D_GLCM_IMC2 | 0.07 | 1.65 |
|  | LoG-sigma-5-0-mm-3D_GLCM_IMC2 | 0.03 | 0 |
| **Shape based (1)** | Original_Shape_MajorAxisLength | 0.04 | 0 |
| **GLSZM (2)** | Wavelet-HLH_GLSZM_SZN | 0.01 | 0 |
|  | LoG-sigma-4-0-mm-3D_GLSZM_GLNN | 0.11 | -0.05 |
| **GLDM (2)** | Wavelet-HLH_GLDM_LDHGLE | 0 | 1.20 |
|  | Wavelet-HHH_GLDM_LDHGLE | 0 | 0.53 |
| **GLRLM (1)** | Original_GLRLM_HGLRE | 0.04 | 0 |

Note: The features were first listed based on their types. Within each type, the features were grouped by their associated pre-processing methods. The value of DT denotes the decrease of Gini index when such feature was selected in the DT model. A high DT value suggests more significant influence in differentiating ALK mutated status and non-ALK mutated status. It is non-directional. The value associated with the LR is the beta coefficient. Since all features were rescaled before entering selection procedure, these coefficients are equivalent to normalized LR coefficients. It is directional. A feature with a higher positive value suggests a stronger association with ALK-rearranged tumours.

Reference

1. Indrayan A, Kumar R, Dwivedi S. A simple index of smoking. *COBRA Reprint Series* [Internet]. (2008 09 September 2019); Paper 40. Available from: <https://www.researchgate.net/publication/272482577>.
